# Enhancing malaria-in-pregnancy monitoring: stakeholder experiences and data integration into the BornFyne-PNMS digital platform in Cameroon

**DOI:** 10.1136/bmjgh-2025-020527

**Published:** 2026-07-06

**Authors:** Armel Mekountchou Tasségning, Veronica Shiroya, Valery Ngo, Franck Wanda, Victor Ndiforchu, Nkengfac Fobellah, Marcelin Ateba, Dominique Bomba, Alice Tabebot, Zacchaeus Ebongo, Donaldson F Conserve, Mwenya Kasonde, Tigest Tamrat, María Barreix, Rosemary K Muliokela, Nasser Bangai Tizi, Sanni Yaya, Miriam Nkangu

**Affiliations:** 1 Health Promotion, Health Promotion Alliance of Cameroon, Yaoundé, Cameroon; 2 Heidelberg Institute of Global Health, University Hospital of Heidelberg, Ruprecht-Karls University of Heidelberg, Heidelberg, Baden-Wuerttemberg, Germany; 3 Centre for Prevention and Digital Health, Medical Faculty Mannheim of Heidelberg University, Mannheim, Baden Wuerttemberg, Germany; 4 Health Promotion Alliance of Cameroon, Yaoundé, Cameroon; 5 The International Center for Research, Teaching and Care (CIRES), Akonolinga, Cameroon; 6 Ministry of Public Health Cameroon, Bangem, Cameroon; 7 Ministry of Public Health, La Habana, Cuba; 8 National Malaria Control Program, Ministry of Public Health Cameroon, Yaounde, Cameroon; 9 Department of Family Health (DSF), Ministry of Public Health Cameroon, Yaounde, Cameroon; 10 The George Washington University Milken Institute School of Public Health, Washington, District of Columbia, USA; 11 REACH Global Health Consulting Ltd, London, UK; 12 Department of Sexual, Reproductive, Maternal, Child, Adolescent Health and Ageing, including the UNDP/UNFPA/UNICEF/WHO/World Bank Special Programme of Research, Development and Research Training in Human Reproduction (HRP), World Health Organization, Geneva, Switzerland; 13 Department of Health Promotion, Ministry of Public Health Cameroon, Yaounde, Cameroon; 14 The George Institute for Global Health, Imperial College London, London, UK; 15 Bruyere Health Research Institute, Ottawa, Ontario, Canada

**Keywords:** Global Health, Health policy, Maternal health, Public Health, Health systems

## Abstract

Malaria in pregnancy (MiP) is a significant public health concern in high-burden malaria countries and a key predictor of high-risk pregnancies in Cameroon. One of the challenges of the National Malaria Control Programme (NMCP) in Cameroon is to generate reliable, timely, accurate and actionable data to inform decision-making, effective monitoring and planning. Discussions with the NMCP at the Ministry of Public Health (MoPH) identified poor data quality, lack of clear guidelines, manual data processing at the district level and delays in feedback on data as key bottlenecks in effective implementation of malaria control in Cameroon. This paper documents the data gaps for MiP identified by the digital health platform, BornFyne, project team in collaboration with the MoPH. We outlined lessons learnt in identifying and defining MiP-related indicators through stakeholder engagement across various levels, and identifying priority MiP elements for health facility registers for integration into the BornFyne digital platform. These lessons and insights will provide valuable guidance for other countries looking to incorporate malaria-related content into their antenatal care packages and/or to integrate similar content into their digital health platforms, and suggestions for strengthening the WHO digital adaptation kit MiP content.

**WHAT IS ALREADY KNOWN ON THIS TOPIC**

- Malaria in pregnancy (MiP) is a leading contributor to high-risk pregnancies in Cameroon. However, the National Malaria Control Programme faces challenges in capturing reliable, timely and actionable MiP data due to poor data quality, unclear guidelines, manual data processing at district levels and feedback delays.

**WHAT THIS STUDY ADDS**

- This practice paper demonstrates how digital platform teams can collaborate with national and district-level stakeholders to define and prioritise indicators for integration into routine health facility registers and digital tools. The participatory process, benchmarked against best practices, strengthened stakeholder alignment and ensured that the selected indicators reflected real-world needs and aligned with national reporting requirements

**HOW THIS STUDY MIGHT AFFECT RESEARCH, PRACTICE OR POLICY**

- The lessons from Cameroon provide practical guidance for countries seeking to improve MiP data through digital platforms. This experience contributes to the global dialogue on how to adapt and strengthen the malaria content of the WHO Digital Adaptation Kits for antenatal care. The paper emphasise the importance of country-led digital transformation initiatives and cross-sector collaboration in improving MiP outcomes and health data systems.

## Introduction

 Malaria in pregnancy (MiP) is a critical public health concern in sub-Saharan Africa, where it has been linked to a high prevalence of low birth weight, a major contributor to infant mortality.^1^,^3^ The highest prevalence of malaria exposure during pregnancy in 2022 was observed in Central and West Africa (40.1% and 39.3%, respectively),^4^ where many neonates were born with low birth weight, particularly in cases where pregnant women (PW) did not receive intermittent preventive treatment during pregnancy (IPTp).^2^ In 2023, an estimated 263 million malaria cases were reported globally across 83 endemic countries, primarily in the African region, which accounted for 94% of all cases.^2^ Malaria infection poses a substantial risk to PW, leading to adverse outcomes for both the mother and the fetus.^5^

Cameroon is 1 of 11 high-burden, high-impact countries where malaria accounts for 50% of hospitalisations and 19% of health facility deaths.^6 7^ While the proportional morbidity of malaria in the country decreased from 15.53% in 2018 to 10.9% in 2022,^7^ and maternal deaths dropped from 13.1 to 9.0 per 100 000 people in the same period,^7^ the coverage of three doses of IPTp using sulphadoxine-pyrimethamine (IPTp-SP) remains low.^8^ Evidence from Douala revealed persistent vulnerability, with higher malaria prevalence in pregnancy associated with younger maternal age (below 21 years) and not sleeping under mosquito bed nets.^9^ Consequently, the government of Cameroon continues to prioritise the fight against malaria as a public health emergency, as outlined in the country’s 2016–2027 Health Sector Strategy.^6^

Strategies for MiP control in Cameroon include IPTp-SP, insecticide-treated bed nets (ITNs) and case management in accordance with national policies.^10^,^13^ The 2022 Cameroon Malaria Indicator Survey highlighted that 65% of women had at least four antenatal care (ANC) visits, with a higher percentage in urban areas (75%) than in rural areas (56%)^10^; 63% of PW (14–49 years) slept under ITNs, with use being slightly higher among those in rural areas (65%) than in urban areas (60%).^10^ The 82.5% of women with a live birth who received one or more doses of SP/Fansidar for malaria prevention (79.0% in rural area vs 86.6% urban area) dropped to 45.8% for at least three doses (44.9% in rural areas against 46.9% in urban areas).^10^

In 2023, the percentage of PW attending first ANC who received ITNs was 87.4%; and IPTp distribution dropped from 80.5% for the first dose (IPTp1) to 51.3% for the third dose (IPTp3).^7^ Despite government efforts through specific interventions like ITNs, IPTp, malaria screening and treatment of detected cases, progress in reducing incidence and morbidity of malaria among PW has remained inconsistent. For example, from 2018 to 2022, there was a decrease from 2021 (incidence: 90.3%; proportional morbidity: 25.7%) to 2022 (incidence: 87.4%; proportional morbidity: 21.3%).^7^ While malaria remains a major cause of maternal mortality, the recent data from the National Strategic Plan 2024–2028 shows a drop in proportional mortality from 12.15% to 3.6% between 2019 and 2022 among PW.^7^ However, these figures must be interpreted in the context of significant implementation challenges, many of which were spillover effects of the COVID-19 pandemic. These include, for example, supply chain disruptions, delayed procurement of essential commodities and reduced attendance at health facilities, structural barriers such as poor and late ANC attendance, out-of-pocket costs and persistent service gaps, especially in rural areas, may constrain further, the effectiveness of malaria prevention strategies for PW.^7^ These challenges also highlight the urgent need for enhanced data collection, such as through digital systems, for timely monitoring and better planning of interventions.

Despite updated policy promoting monthly dosing of IPTp,^14^ many countries have not updated their ANC registers and/or electronic data platforms (eg, the District Health Information System 2, or DHIS2) to capture routine Health Management Information System (HMIS) data.^1^ Surveys like Demographic and Health Survey and Malaria Indicator Survey also provide data, but are more difficult and expensive to collect and are not used to drive ongoing programme management decisions.^1^ This is because these surveys are periodic and are not designed to provide real-time, timely data needed for daily operational management.

As Cameroon advances a digital health and universal health coverage agenda, its 2026–2030 National Digital Health Strategic framework aims to ensure that data are collected in electronic format and stored for routine follow-up.^15^ The framework aims to generate actionable and reliable data to improve quality of care and reduce morbidity and mortality by improving health outcomes through informed decision-making at all levels.^15^

Generating timely, reliable, accurate and actionable data to inform decision-making and support the monitoring and evaluation of malaria programmes—particularly for MiP—remains a persistent challenge in Cameroon. Recognising the potential of digital platforms to address data gaps and enhance the generation of actionable data, the BornFyne digital health platform was identified by stakeholders to integrate MiP-specific variables into routine ANC.^16^,^18^ This platform was pilot-tested and presented to key stakeholders in 2023, to assess needs and identify strategies to strengthen the collection and use of reliable MiP data.^16^,^18^ This paper outlines the methodology used by the BornFyne team to engage stakeholders in identifying key MiP indicators for integration into an updated version of the BornFyne-PreNatal Management System (PNMS). It emphasises the critical role of stakeholder engagement for improved patient centric care and shares valuable lessons learnt throughout the participatory co-development process. Importantly, we detail the successful and sustained involvement of the Cameroon Ministry of Public Health (MoPH)—from initial planning through implementation, demonstrating how their leadership served as a driving force in identifying the MiP variables for integration. Furthermore, we illustrate how insights from this engagement informed the alignment of MiP data elements with the WHO’s ANC recommendations, as outlined in the WHO ANC Digital Adaptation Kit (DAK).^19^,^22^

## Context: malaria in pregnancy variables within the WHO antenatal care Digital Adaptation Kit data dictionary and BornFyne-PreNatal Management System

The BornFyne-PNMS is a digital platform developed to strengthen reproductive maternal and newborn child and adolescent health services by offering interactive user-friendly features for ANC, family planning and facilitates emergency.^17 23 24^ The ANC module is developed by integrating the WHO’s DAK for ANC content.^18^,^22^ This streamlines workflows while aligning with both national and international ANC guidelines. The DAK is part of the WHO Standards-based, Machine-readable, Adaptive, Requirements-based and Testable (SMART) guideline’s framework,^19^,^22^ serve as operational and software-neutral mechanisms designed to translate WHO guidelines into a standardised format that can be easily integrated into countries’ digital systems.^19^,^22^ BornFyne-PNMS was developed before the launch of the ANC DAK and incorporates a range of variables that are collected during ANC including MiP variables.^16 17^

Table 1 highlights the MiP variables integrated into the BornFyne digital health platform prior to the launch of the WHO DAK in 2021. During ANC, providers are expected to document these MiP variables alongside other routine ANC components into BornFyne platform^16^ (see online supplemental file 1). After the release of the WHO’s DAK, the BornFyne team updated BornFyne’s content to align with DAK content, particularly the data dictionary, ensuring variables included data elements and International Classification of Diseases (ICD) codes.^18^ The DAK’s MiP requirements focuses on counselling and guidance rather than specific indicators or data elements.^22^ This reflects the DAK’s generic design, based on WHO guidelines, which focuses on skilled, facility-based service delivery allowing countries to adapt content for their context or define additional data elements and indicators for their specific need. The DAK’s decision support workflow provides detailed directions for clinical management of malaria in pregnancy, including the administering of IPTp doses 1–3.^22^ To supplement the WHO ANC DAK and ensure a comprehensive set of MiP data and decision support needs of Cameroon, the following process was used: meeting of stakeholders, mapping and engagement using best practices, national data mapping and exploration into routine data collection and processing.

**Table 1:** Existing MiP indicators in the BornFyne-PNMS against WHO ANC DAK malaria content

BornFyne-PNMS			WHO ANC DAK guideline MiP indicators^18^		
Indicator code	Indicator name	Numerator/denominator	Indicator code	Indicator name	Numerator/denominator
None	Proportion of pregnant women having a mosquito net.	Number of pregnant women who have a mosquito net/total number enrolled into the platform.	Present in DAK data dictionary as a counselling element	Does not exist	Does not exist
None	Proportion of pregnant women who ever slept under a mosquito net.	Number of pregnant women who ever slept in a mosquito net/total number enrolled into the platform.	Present in DAK data dictionary as a counselling element	Does not exist	Does not exist
None	Proportion of pregnant women using mosquito net.	Number of pregnant women using mosquito net/total number enrolled into the platform.	Present in DAK data dictionary as a counselling element	Does not exist	Does not exist
None	Does not exist		ANC.IND.6	Pregnant women who received counselling on danger signs (%) during at least one ANC contact	Number of pregnant women who received counselling on danger signs/total number of pregnant women with a first contact
None	Proportion of pregnant women who receive IPTp at ANC.	Number of pregnant women who received IPTp at ANC/total number enrolled into the platform.	ANC.IND.11	Percentage of women who received three doses or more of intermittent preventive therapy for malaria (IPTp) during their last pregnancy	Number of pregnant women given at least three doses of sulfadoxine-pyrimethamine for IPTp/total number of antenatal clients with a first contact

ANC, antenatal care; DAK, Digital Adaptation Kit; IND, indicator; IPTp, intermittent preventive treatment during pregnancy; MiP, malaria in pregnancy; PNMS, PreNatal Management System.

## Stakeholder meeting, mapping and engagement

**Figure 1:**
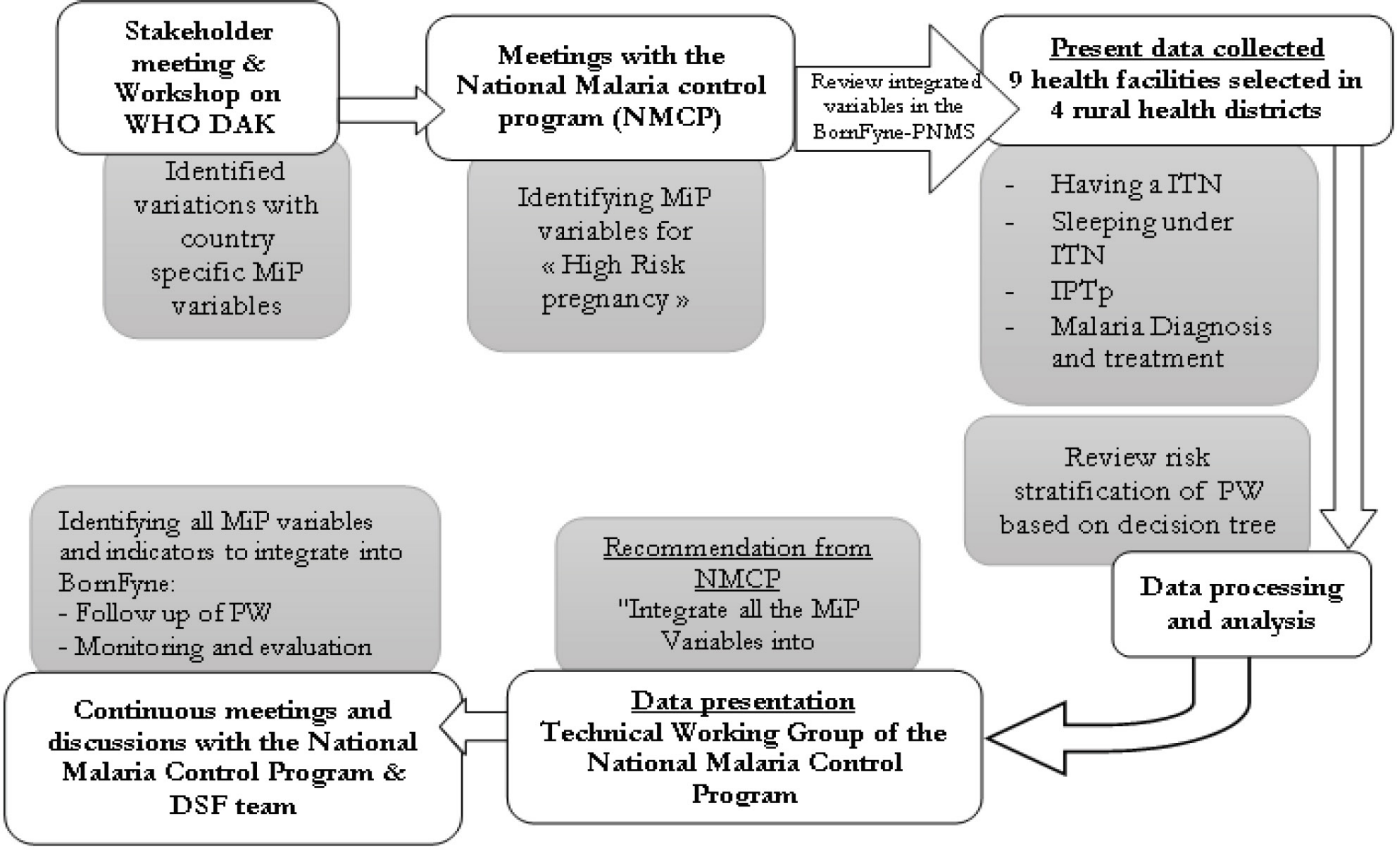
The participatory and inclusive process in identifying MiP variables for an updated BornFyne-PNMS. DAK, Digital Adaptation Kit; DSF, Department of Family Health; IPTp, intermittent preventive treatment during pregnancy; ITN, insecticide-treated bed nets; MiP, malaria in pregnancy; NMCP, National Malaria Control Programme; PNMS, PreNatal Management System; PW, pregnant women.

Figure 1 maps the overall process with the main results. In August 2023, in partnership with the Department of Family Health at the MoPH, the team organised a stakeholder meeting to present the midterm results of the BornFyne project transition to scale phase 1, implemented in four health districts (Akonolinga, Ayos, Bangem and Tiko) across nine health facilities—including the MiP results for the four variables listed in table 1 above^16^ and online supplemental file 1. The meeting engaged both national and international partners.

A total of seven meetings including consultations were held. Participants included representatives from key governmental, international and community organisations. Specifically, the second central-level stakeholder meeting included over 25 institutions, such as EVIHDAF, WHO, UNICEF, United Nations Population Fund, United States Agency for International Development and various MoPH departments. Participants were selected through a snowball sampling approach led by the directorate and MoPH, where focal persons recommended key informants across departments. This ensured sectoral representation, particularly from malaria programmes, maternal health, data systems and community-level actors. While we aimed for inclusivity, we acknowledge that some civil society voices may have been underrepresented due to the nature of institutional referrals.

After the stakeholder meeting, the BornFyne team met two times with experts from the National Malaria Control Programme (NMCP) at the MoPH to explore opportunities for managing malaria data among PW through the BornFyne-PNMS platform. Following these discussions, the team was invited to present partial results on malaria during the seventh Technical Working Group meeting on malaria in pregnancy, held in February 2024. During the meeting, stakeholders from the NMCP recommended that the BornFyne team engage with malaria programme officials, directors and partners to collect additional feedback and strengthen the application aimed at improving IPT3 coverage.

Subsequently, consultations were held with key informants at the NMCP, identified by the MiP focal person. The BornFyne team visited four NMCP departments—Prevention, Integrated Vector Malaria Programme, Data Management and Malaria Case Management, to gather insights on programme needs and explore digital solutions to enhance MiP interventions. Findings from these consultations were presented at the subregional level during the second quarterly meeting of the Roll Back Malaria(RBM) MiP Working Group Central Africa Network in July 2024. Following this, the BornFyne team was invited to the MiP Working Group Annual Meeting in September 2024, in Kenya, where the results were again presented. This process culminated in the development of a concept note for validation of the findings. (See table 3 and the remaining variables are listed in online supplemental table 1). A review of stakeholder recommendations was conducted, challenges related to generating malaria data during pregnancy were discussed and opportunities for integrating MiP variables into the BornFyne platform were explored.

## Benchmarking stakeholder engagement against best practices

In addition to contemporary case studies, the stakeholder engagement strategy reflects approaches recommended in foundational literature. Stakeholders were engaged from the design phase in 2022, ensuring early input before platform deployment.^25^ The process was inclusive, involving policymakers, frontline health workers and end beneficiaries, which is widely recognised as essential for relevance and sustainability.^26^ Engagement was structured through six central-level and district-level meetings over 14 months, creating regular feedback loops and enabling iterative adaptation of the platform—an approach particularly important for complex digital health initiatives.^27^ Finally, stakeholders acted as active co-design partners, directly shaping priorities and platform features (eg, malaria in pregnancy variables). This participatory model aligns with the gold standard in digital health research, fostering ownership, empowerment and increased likelihood of adoption.^28^

## Findings from the stakeholder consultations in identifying malaria in pregnancy variables

The BornFyne team engaged MoPH experts to review how MiP data processing is coordinated across different levels of the health system. This was in order to define specific needs and MiP indicators for integration into the BornFyne-PNMS. Data for monitoring and evaluation of malaria programmes is essentially from: health facility registers to the National Health Information System digital storage, the DHIS2, through health facilities surveys, health districts and national meeting reports (Cameroon National RBM coordination, etc), evaluation report of health districts, periodic reports of the NMCP (annual, biannual, quarterly), National Household Surveys (Malaria Indicator Survey, Multiple Indicator Clusters Survey, Demographic and Health Survey, Malaria Behaviour Survey) and the global malaria report.^22^

Discussions with MoPH experts revealed redundancy in data collection across departments for key variables such as IPTp, ITNs and malaria treatment. This overlap underscored the need for a more centralised and integrated platform. While the redundancy reflected a point of disagreement with current practices, it also provided a strong argument for aligning stakeholders around the value of a unified system.

A key recommendation from international partners was for the team to collaborate with the national malaria programme to explore ways to improve data collection for MiP. In response, a workshop was conducted with health providers from selected health districts to assess the DAK variables for malaria against the health facility register. Mapping of key stakeholders for consultations was conducted in collaboration with the MiP focal person at the NMCP within the MoPH. Our findings highlighted the following:

### Current routine data processing for malaria in pregnancy in Cameroon

Guided by the WHO Global Technical Strategy for Malaria 2016–2030 (2015) and the Framework for malaria elimination (2017), Roll Back Malaria working groups and other stakeholders established core routine indicators for malaria-endemic countries to assess programme performance.^1^ In Cameroon, each level of the health system structural organisation can add contextual indicators to those provided by the NMCP, ensuring alignment with WHO recommendations.^29 30^ However, there is no specific monitoring of how these are added and/or harmonised.

Routine individual MiP data in Cameroon are essentially collected using non-digital collection tools (registries and/or forms) at: peripheral level (community and health facilities) and health facilities at intermediary and central level of the health system. Data collected are aggregated in monthly activity report (MAR) on paper formats and stored at health facilities with trained personnel and district health office in the DHIS2 digital database^31^,^33^ (see table 2). Data analysis and use for decision-making are done at all levels of the health system.^7 33^ Data collected by community health workers (CHW) are transmitted to the health facility, then the health district.^31^,^33^ After data are entered into the DHIS2, they are transmitted to regional and central levels for quality assessment, analysis, interpretation and usage.^33^ At the end of the process, physical data collection tools (registry, forms, etc) are kept for at least 10 years in chromos and shared according to the period of the year; while electronic archiving is undertaken on external devices (hard drives, flash drive) for digital data.^31^ Table 2 below indicates the way routine data are processed from the peripheral to the central level.

**Table 2:** General routine data collection and processing tools for malaria in Cameroon

Setting	Collector	Data processing		
		Collection	Aggregation	Storage
Community	CHW	Registers, form	Monthly activity report for CHW	No electronic storage, paper register.
Health facilities (public and private)	Health provider	Health facility registers (outpatient, hospitalisations, emergency consultations, ANC, laboratory), inputs forms for anti-malaria drugs, forms for ITNs and sulfadoxine-pyrimethamine distribution, forms for data synthesis; data analysis canva National malaria programme collection form	Monthly activity report of regional, central, general and referral hospitals	DHIS2*
Health area	Head of the health area	Health facilities and CHW registers and other data collection forms provided to CHW	Monthly activity report of regional, central, general and referral hospitals	DHIS2*
Health districts	Malaria focal point Stocks management focal point	–	–	DHIS2 Dashboard Data analysis canva

* If trained personnel to fill the DHIS2 is available at the health facility, as they are normally found at the health district.

ANC, antenatal care; CHW, community health workers; DHIS2, District Health Information System 2; ITN, insecticide-treated bed nets.

### Streamlining data collection process

During discussions with MoPH experts, it was noted that data requirements overlapped across departments, particularly for indicators such as IPTp coverage, ITN usage and malaria treatment. Similar redundancies were observed in reporting on CHW activities at community level (communication, education, community assessments). This highlighted the need for a more centralised and integrated platform, capable of aggregating data accordingly and disseminating tailored outputs according to decision-making priorities. Such a platform must also facilitate interoperability with existing HMIS to enhance the quality-of-care delivery and improve data in an accessible and actionable format. Strengthening these systems is essential for informing policies and programmatic intervention strategies towards reducing maternal mortality and achieving Universal Health Coverage.^16^

### Challenges and limitations of malaria in pregnancy variables in routine health information system

Cameroon as other countries in Sub Saharan Africa still faces difficulties concerning data quality assurance, thus constituting a gap in the decision-making process.^31 33 34^ Each month, routine data from health facilities is submitted to the district level by the 5th day, then forwarded to the regional level by the 10th day and finally to the national level by the 15th day—resulting in a minimum 15-day delay from the health facility (or community) to the national level.^31 33^ In the existing non-automated data processing system, the acknowledgement of data receipt, feedback after analysis and interpretation and the generation of summary data tables at each level are not timely or systematic.^31^

The DHIS2 is scaled up at national level in Cameroon to boost harmonisation; but their usage is not always effective at peripheral levels and some health regional delegation.^31 34^ Data quality is also affected by incompleteness, delays in report submission and low data consistency.^24^ These observations triggered the development of a procedure manual for malaria data analysis and utilisation in 2019.^31^ Despite the efforts for data processing, real data harmonisation starts only after individual collection from clients, when they are manually aggregated and digitally stored. There are no defined questions, nor guidelines for individual level disaggregated malaria data collection from PW; and the process from collection to storage, through aggregation is not automated. This is the responsibility of trained health facility workers or district supervisors.

Regular ANC contacts offer an opportunity for consistent malaria data collection at health facilities. However, the risk of poor data collection, errors during aggregation and long delays in notifying missing data persist. The variables and indicators identified through the consultation process with focal persons from the malaria programme are presented in table 3 below. The NMCP has requested the BornFyne team to integrate these variables into the BornFyne platform. However, since the variables are in aggregated form, the BornFyne research team and the NMCP of the MoPH will need to define specific questions before they can be integrated. Table 3 summarises the data that are collected in health facility registers, monthly activity reports and the DHIS2 system for malaria, alongside existing guidance from the DAK for ANC concerning MiP. Since the BornFyne ANC module aligns with the DAK standards and format, the questions to be identified and validated will need to be coded with corresponding data elements and ICD codes (where applicable).

**Table 3:** MiP data elements collected at peripheral level at various settings in the current version of BornFyne-PNMS versus the other reporting tools

	BornFyne-PNMS malaria variables during ANC Data reported in the digital PNMS	Community by CHW in MAR Data reported in MAR for CHW	Health facility registers during ANC Data reported in MAR and national malaria programme collection sheet	WHO digital adaptation kit for antenatal care (WHO ANC DAK)^18^	DHIS2 Data reported in the digital DHIS2 form
Identification/ registration	Date, region, health district, health area, health facility, collects GPS coordinates, education, employment, income, cell phone ownership among women,* internet use among women.*	Date, region, health district, health area, health facility GPS coordinates Hygiene and sanitation data† for identifying malaria risk factors^8^ (drinking water; sanitation; household composition, education among women aged 15–49 years, cell phone ownership among women aged 15–49 years, internet use among women).	Date, region, health district, health area, health facility	Available in DAK under patient profile **Registration:** Unique identification, first name, last name, contact date, date of birth, age, address, mobile phone number (optional), woman want to receive reminders during pregnancy? (optional), alternative contact’s name, alternative contact’s phone number, co-habitants. **Profile:** Highest level of education achieved, occupation, GA, source of GA, number of pregnancies (gravida), whether last live birth was preterm, past pregnancy complications, substance use during past pregnancy, allergies, past surgeries, existing chronic health conditions, tetanus toxoid-containing vaccine immunisation history, current medications, daily caffeine intake, current alcohol and/or substance use, tobacco use or exposure, partner HIV status **ANC contact:** Registration (see registration), pregnancy confirmed? reason for coming to facility, specific health concern(s), danger signs?	Date, region, health district, health area, health facility
IPT for pregnant woman	No. of pregnant women in ANC 1, 2, 3, 4 up to 8 No. of pregnant women on IPTp (non-specific)	None ANC attendance† IPTp2 and above distributed at community level† (WHO recommendation^32^ but not yet applicable in this context)	No. of pregnant women in ANC 1, 2, 3, 4 No. of pregnant women on IPTp 1, 2, 3, 4	Percentage of pregnant women with first ANC contact in the first trimester (before 12 weeks of gestation) Percentage of pregnant women with at least four ANC contacts Percentage of pregnant women with a minimum of eight antenatal care contacts Percentage of women who received three doses or more of intermittent preventive therapy for malaria (IPTp) during their last pregnancy	No. of pregnant women in ANC 1, 2, 3, 4 No. of pregnant women in ANC 1, 2, 3, 4
Communication for behaviour change/social mobilisation	None	No. of households visited. No. of people sensitised through home visits, No. of shared printed material Knowledge/counselling on the use and maintenance of ITNs† Reasons for not taking IPTp†	None	Pregnant women who received counselling on danger signs (%) during at least one ANC contact malaria-endemic setting: Whether the setting is a malaria-endemic setting Deworming and malaria prophylaxis	No. of households visited No. of people sensitised through home visits, No. of shared printed material
Morbidity, mortality and case management	No. of pregnant women with LLINs No. of pregnant women using LLINs Frequency of using LLINs No. of maternal death from any cause No. of neonatal death	No. of reported fever cases No. of tested fever cases No. of diagnosed simple malaria cases No. of treated simple malaria cases No. of referred severe malaria cases No. of maternal death No. of neonatal death	No. of pregnant women with LLINs No. of suspected malaria cases No. of tested suspected malaria cases No. of confirmed malaria cases No. of treated malaria cases No. of death from any cause No. of death from confirmed malaria	Number of IPTp2 and above distributed^32^	No. of reported fever cases No. of referred severe malaria cases No. of pregnant women with LLINs No. of suspected malaria cases No. of tested suspected malaria cases No. of confirmed malaria cases No. of treated malaria cases No. of death from any cause No. of death from confirmed malaria No. of maternal death No. of neonatal death

* Collected by CHW during household visit.

† Not routinely collected but identified during discussion with focal points at the Cameroon national malaria programme as important variables to consider and introduce in BornFyne System.

ANC, antenatal care; CHW, community health worker; DAK, Digital Adaptation Kit; DHIS2, Digital Health Information System 2; GA, gestational age; IPTp, intermittent preventive treatment during pregnancy; ITN, insecticide treated net; LLIN, long-lasting insecticidal net; MAR, monthly activity report; MiP, malaria in pregnancy; No, number; PNMS, PreNatal Management System; PW, pregnant woman.

To facilitate then next steps, the team has engaged the WHO ANC DAK development team for technical assistance. In line with the DAK approach of adaptation and tailoring to each setting and should reflect national ANC guidelines including MiP. As such, all variables identified by the NMCP are relevant to the Cameroonian context and will be integrated into BornFyne. For example, variables such as fever cases detected by CHWs, IPTp doses distributed by CHWs and environmental hygiene activities currently fall outside the ANC DAK’s scope.

## Monitoring and management of malaria during pregnancy in Cameroon

Monitoring and managing MiP is crucial for the health of both the pregnant woman and fetus as a positive malaria diagnosis denotes a high-risk pregnancy. MiP is considered severe malaria and fever or other relevant symptoms in pregnancy should always be considered an emergency or a danger sign that should be managed at health facility with the recommended treatment made of initial parenteral dose of artesunate or artemether; tocolytics will be administered in case of severe uterine contractions.^13^ Early diagnosis is crucial for effective management and improved maternal and fetal outcomes.^35^

Together with training of community stakeholders, and awareness-raising of beneficiary populations, the provision of routine antimalarial care during pregnancy made of supply with rapid diagnosis tests, antimalarial drugs, distribution of ITNs and IPTp administration during ANC visits, is integrated into care packages recommended to health facilities.^4^ Mass ITNs distribution campaigns and seasonal malaria chemoprevention campaigns are organised to strengthen routine malaria control.^4 12^ The malaria vaccine, introduced in Cameroon, has been a strong WHO recommendation since 2021. Cameroon began using it in January 2024 to prevent *Plasmodium falciparum* malaria.^29 36^ In parallel, a social and behaviour change strategy is also underway to support the implementation and sustenance of current interventions.^37 38^

## Lessons learnt from the field

### Collaboration of BornFyne team with national malaria programme in Cameroon

The collaboration provided critical insights into data collection, revealing gaps and redundancies in how MiP health data are captured, stored and reported. It also shed light on operational challenges that compromise data quality and management. These findings (see table 4 and online supplemental table 2) will guide the integration of MiP indicators into the BornFyne platform and inform strategies to address the gaps. All indicator reports are harmonised using the MAR, with versions tailored for healthcare providers and CHWs. However, issues persist: the lack of a standardised guide, time-intensive reporting, multiple data sources (eg, registries, patient files) and uncertainty about data completeness. Digitisation remains a key gap. Healthcare providers and district personnel need training in DHIS2. While DHIS2 reporting is typically done at the district level, health facilities with trained personnel can enter data directly. Otherwise, they must submit the MAR to the district for DHIS2 reporting. In the MAR, they record the number of PW under 15 years and also the total number of women received, organised by age-groups. The health unit responsible for harmonising the data verifies these figures by manually counting entries in the health facility register before completing the MAR. The report is then sent to the district, which inputs the data into DHIS2. This process is time-consuming and delays reporting. While the 2019 documentation provides a solid framework, integrating digital systems—such as BornFyne—could significantly streamline data collection and reduce delays.

**Table 4:** Identified malaria variables/indicators during consultations and discussions with focal points for national malaria programme

Department at the national malaria programme	Main indicators identified during consultations by malaria programme to be integrated into BornFyne-PNMS			Priority needs	How can community level help?
	Indicators	Method of calculating the indicators (numerator/denominator)	WHO ANC DAK Indicator=(numerator)/(denominator)		
Prevention	ANC attendance rate (%)	Number of PW received in ANC1/number of pregnant women expected over the period (pregnant population per month multiplied by the number of months)	**Percentage of pregnant women with first ANC contact in the first trimester (before 12 weeks of gestation**)=(number of pregnant women who had their first ANC contact before 12 weeks (facility level))/total number of antenatal clients with a first contact	Assessment of frequency for ANC attendance identify the number and proportion of women who start IPTp at the 13th week of pregnancy. Assess the IPTp3 uptake among PW Determine the prevalence of malaria among pregnant women in terms of number of episodes	CHW can check the reasons for not taking IPTp (stock-out, missing visit, …)
	ANC 4 attendance (%)		**Percentage of pregnant women with at least four ANC contacts**=(number of pregnant women with four ANC contacts)/(total number of antenatal clients with a first contact)		
	ANC 8 attendance (%)		**Percentage of pregnant women with a minimum of eight antenatal care contacts**=(number of pregnant women with eight ANC contacts)/(total number of pregnant women with a first contact)		
	IPTp3 coverage (%)	Number of pregnant women who received IPTp3/number of pregnant women received in ANC1	**Percentage of women who received three doses or more of intermittent preventive therapy for malaria (IPTp) during their last pregnancy**=(number of pregnant women given at least three doses of sulfadoxine-pyrimethamine for IPTp)/(total number of antenatal clients with a first contact)		
	Counselling on danger signs (%)		**Pregnant women who received counselling on danger signs (%) during at least one ANC contact**=(number of pregnant women who received counselling on danger signs/total number of pregnant women with a first contact)		
	Behavioural counselling for the pregnant woman on how they individually prevent their exposure to malaria?				
	ANC 2, ANC 3, ANC 4 and above loss to follow-up rate (%)				
	IPTp 2 IPTp3, IPTp 4 and above loss to follow-up rate (%)				
	Proportion of loss to follow-up (%)				
Integrated vector malaria programme	LLIN distribution rate (%)	Number of LLINs routinely distributed to PW/number of PW received in ANC 1		Determine the number and proportion of women who receive and effectively use the LLINs	Assessing KAP of women regarding usage of LLINs (check the instalment, usage and maintenance of the LLINs advice on good use and maintenance of LLINs) Identify via the DHIS 2 (MAR) the main communication channels used to inform PW on ‘How to use MN’ (healthcare provider; CHW, neighbour…)
	Routine LLIN performance (%)	Number of LLINs routinely distributed to pregnant women/number of LLINs to be routinely distributed during the period considered (performance framework)			

Note: Empty cells indicate the numerator, and denominator was not provided and will be defined by the team as part of the next steps.

* Indicates it is present in DAK and the content has been integrated into BornFyne content with corresponding ICD codes. Any other element without an asterisk implies the team is yet to identify and define the questions and validate with the NMCP and test it before it is integrated into the BornFyne content.

ANC, antenatal care; ASAQ, artesunate-amodiaquine; BS, blood smear; CHW, community health worker; DAK, Digital Adaptation Kit; DHIS2, Digital Health Information System 2; ICD, International Classification of Diseases; IND, indicator; IPTp, intermittent preventive treatment during pregnancy; KAP, knowledge attitude practice; LLIN, long-lasting insecticide net; MAR, monthly activity report; MiP, malaria in pregnancy; NMCP, National Malaria Control Programme; PNMS, PreNatal Management System; PW, pregnant woman; RDT, rapid diagnostic test.

Improving communication between CHWs and providers is essential. CHWs collect household-level data, such as IPTp uptake and adolescent pregnancies—often not reviewed by facility staff. CHWs conduct weekly home visits and submit MARs monthly, but their reports are sometimes overlooked, limiting their utility in patient care. Further, risk factor data, currently collected triennially, should be gathered more frequently and integrated into community reports for timely intervention. Strengthening digital tools like BornFyne and fostering collaboration between CHWs and healthcare providers can significantly improve data quality, reduce delays and enhance service delivery.

## Transparency and alignment with country’s digital strategic framework

In Cameroon, the recently launched National Health Digital Strategic Plan serves as the official reference framework guiding all digital health interventions, and a decisive contribution to achieving Universal Health Coverage.^15^ The presentation of the BornFyne-PNMS data for MiP at stakeholder forums and during technical working group meetings of the NMCP initiated important discussions, centred on identifying which specific MiP variables could be feasibly integrated into the BornFyne digital health platform. Being transparent at this stage triggered the vision and key points focusing on local priorities aligned with the national digital health agenda and resulted in a formal resolution by the national malaria programme recommending that the BornFyne team update the BornFyne-PNMS, for improved IPTp 3 coverage and enhancing the follow-up of PW.^16^

## The role of local leadership to drive coordination efforts

Strong local leadership greatly enhanced coordination and follow-up. Direct engagement with MoPH experts and familiarisation with the BornFyne-PNMS platform fostered trust, strengthened collaboration and improved stakeholder communication. This involvement helped MoPH experts clearly see how BornFyne-PNMS aligns with national malaria programme objectives, bridging international recommendations with local needs. The collaboration led to a joint presentation by the BornFyne team and the NMCP at a 2024 conference in Nairobi, Kenya. BornFyne team also serves as a technical partner to NMCP and actively participates in Central Africa’s quarterly and national coordination meetings to align efforts.

## Implications for practice

Observations from the consultation process revealed inconsistencies in data collection across health districts, as indicators are defined centrally, but the actual questions used are determined by health providers or district teams. This lack of a standardised approach affects data quality and comparability. To address this, the BornFyne team will collaborate with the NMCP to conduct a workshop aimed at harmonising data collection by developing standardised questions for use across all health facilities. This initiative, undertaken with NMCP, the DFH and in consultation with the WHO Department of Sexual and Reproductive Health and Research, lays the groundwork for integrating MiP indicators into the WHO ANC DAK data dictionary. The team will also engage regional MiP focal persons to review and validate the proposed data variables and questions.

This practice paper focus is on the methodology for engaging stakeholders to identify and integrate MiP indicators into the BornFyne platform. It emphasises the indicators identified during the consultations with the NMCP, which are primarily focused on process-oriented measures like IPTp doses received, ITN distribution and fever cases detected. While these are crucial for monitoring programme implementation, they do not directly capture the adverse birth outcomes—such as preterm birth and low birth weight—that are the ultimate goals of MiP control efforts. Although the consultations did not provide a reason for excluding outcome indicators, the findings underscore the need for continued collaboration to define and integrate these measures into the BornFyne platform.

Next steps include facilitating a workshop to define and validate questions, identifying corresponding ICD codes and conducting a pilot in select health facilities before full integration into the BornFyne-PNMS platform.^22^ These steps have also been shared with WHO, which responded with guidance on tailoring malaria indicators within national digital health systems. WHO’s approach includes defining indicators with clear numerators and denominators, designing appropriate data collection questions and applying a structured digital adaptation process. While the current WHO ANC DAK focuses on facility-based interventions,^22^ community-level indicators such as CHW-detected fever or IPTp distribution are listed as ‘Not Applicable’ and will be considered in future DAKs targeting community health.

## Implications for policy

In our previous work integrating the WHO DAK, we noted the absence of normative ANC guidelines at health facilities, guidelines that should, at a minimum, include key MiP indicators.^16^ Thus, the BornFyne team recommended the need for updated ANC guidelines for health facilities by contextualising and adapting the DAK for ANC guidelines. While Cameroon may be implementing the WHO’s eight-contact ANC model, there are no standardised documented ANC guidelines at the facility level to confirm this. Given the high malaria burden and its impact on maternal and neonatal health, we recommend that WHO integrate MiP-specific variables and ICD codes into the DAK data dictionary—or develop a dedicated DAK for malaria. This would enhance consistency, data interoperability and country-level adaptation. As DAKs evolve based on global experiences, the balance between comprehensive data and practical feasibility must be carefully considered.

## Conclusion

Integrating MiP indicators into national digital health systems requires strong national commitment, clear guidelines and systematic capacity building. Key lessons from our experience in Cameroon highlight the importance of standardised data collection, deeper integration of community health workers through digital tools and alignment with the DAK for global consistency. The DAK proved instrumental in accelerating digitalisation and harmonising data systems. We recommend that the WHO consider expanding or creating a malaria-specific DAK to support countries further. Insights from country-level adaptations via initiatives like Cameroon’s can strengthen global guidance and improve maternal health outcomes through effective digital health integration.

## Supplementary material

10.1136/bmjgh-2025-020527 online supplemental table 1

10.1136/bmjgh-2025-020527 online supplemental table 2

## Data Availability

All data relevant to the study are included in the article or uploaded as supplementary information.
