# Supplementary material for: Enhancing malaria-in-pregnancy monitoring: stakeholder experiences and data integration into the BornFyne-PNMS digital platform in Cameroon
Source: BMJ Glob Health. 2026 Jul 6;11(7):e020527. doi: 10.1136/bmjgh-2025-020527 (PMC13343098; doi:10.1136/bmjgh-2025-020527)
Supplement: online supplemental table 1 [file bmjgh-11-7-s001.pdf]

Supplemental File 1: BornFyne-PNMS Malaria in pregnancy pilot data

Selected demographic characteristics of the enrolled women and malaria variables collected within the BornFyne-PNMS platform

Variables	Akonolinga (N=55) n (%)	Ayos (N=60) n (%)	Bangem (N=67) n (%)	Tiko (N=261) n (%)	Total 443
**Residence**					
Rural	4 (7.27)	7 (11.67)	23 (34.33)	14 (5.36)	**48**
Semi–urban	46 (83.64)	4 (6.67)	27 (40.30)	223 (85.44)	**300**
Urban	5 (9.09)	49 (81.67)	17 (25.37)	24 (9.20)	**95**
**Age [mean (sd)]**	**25.34 (6.53)**	**27.40 (6.57)**	**27.83 (6.06)**	**27.51 (5.81)**	**27.27 (6.07)**
**Marital Status**					
Divorced	0 (0.00)	0 (0.00)	4 (5.97)	2 (0.77)	**6**
Monogamy	10 (18.18)	16 (26.67)	42 (62.69)	165 (63.22)	**233**
Polygamy	3 (5.45)	2 (3.33)	5 (7.46)	4 (1.53)	**14**
Single, never married	42 (76.36)	42 (70.00)	16 (23.88)	89 (34.10)	**189**
Widowed	0 (0.00)	0 (0.00	0 (0.00	1 (0.38)	**1**
**Education**					
No formal education	1 (1.82)	1 (1.67)	9 (13.43)	4 (1.53)	**15**
Primary	7 (12.73)	14 (23.33)	13 (19.40)	25 (9.58)	**59**
High school	17 (30.91)	14 (23.33)	13 (19.40)	81 (31.03)	**125**
Secondary	19 (34.55)	22 (36.67)	21 (31.34)	92 (35.25)	**154**
University	11 (20.00)	9 (15.00)	11 (16.42)	55 (21.07)	**86**
Other	0 (0.00)	0 (0.00)	0 (0.00)	4 (1.53)	**4**
**Income**					
30,000 CFA and below	6 (10.91)	44 (73.33)	29 (43.28)	59 (22.61)	**138**
30,001 - 70,000 CFA	5 (9.09)	10 (16.67)	25 (37.31)	102 (39.08)	**142**
70,000 CFA and above	14 (25.45)	2 (3.33)	4 (5.97)	26 (9.96)	**46**
No income	30 (54.55)	4 (6.67)	9 (13.43)	74 (28.35)	**117**

Variables	Akonolinga, (N=55) n (%)	Ayos, (N=60) n (%)	Bangem, (N=67) n (%)	Tiko, (N=261) n (%)	Total 443
**IPTp**					
No	12 (21.82)	19 (31.67)	20 (29.85)	55 (21.07)	**106**
Yes	43 (78.18)	41 (68.33)	47 (70.15)	206 (78.93)	**337**
**Do you have a Mosquito net?**					
No	12 (21.82)	12 (20.00)	24 (35.82)	36 (13.79)	**84**
Yes	43 (78.18)	48 (80.00)	43 (64.18)	225 (86.21)	**359**
**Have you slept under a Mosquito Net before?**					
No	6 (10.91)	4 (6.67)	24 (35.82)	11 (4.21)	**45**
Yes	49 (89.09)	56 (93.33)	43 (64.18)	250 (95.79)	**398**
**When last you slept under Mosquito Net**					
Do not Remember	4 (7.27)	4 (6.67)	3 (4.48)	19 (7.28)	**30**
Every Day	0 (0.00)	1 (1.67)	14 (20.90)	1 (0.38)	**16**
Last Month	4 (7.27)	0 (0.00)	23 (34.33)	6 (2.30)	**36**
Last Week	1 (1.82)	3 (5.00)	13 (19.40)	29 (11.11)	**43**

Not Started	1 (1.82)	2 (3.33)	0 (0.00)	10 (3.83)	**13**
Some Day	0 (0.00)	0 (0.00)	3 (4.48)	6 (2.30)	**9**
Yesterday	45 (81.82)	50 (83.33)	11 (16.42)	190 (72.80)	**296**
