# Supplementary material for: Enhancing malaria-in-pregnancy monitoring: stakeholder experiences and data integration into the BornFyne-PNMS digital platform in Cameroon
Source: BMJ Glob Health. 2026 Jul 6;11(7):e020527. doi: 10.1136/bmjgh-2025-020527 (PMC13343098; doi:10.1136/bmjgh-2025-020527)
Supplement: online supplemental table 2 [file bmjgh-11-7-s002.pdf]

Supplemental table 2: Identified malaria variables/indicators during consultations and discussions with focal points for national malaria program

**Department at the National malaria program**	**Main indicators identified during consultations by malaria program to be integrated into BornFyne-PNMS**			**Priority needs**	**How can community level help?**
	**Indicators**	**Method of calculating the indicators** (Numerator / *Denominator*)	**WHO ANC DAK Indicator =** [Numerator]/[Denominator]		
**Prevention**	*ANC Attendance rate (%)	Number of PW received in ANC1 / *Number of pregnant women expected over the period (pregnant population per month multiplied by the number of months)*	**Percentage of pregnant women with first ANC contact in the first trimester (before 12 weeks of gestation)** = [Number of pregnant women who had their first ANC contact before 12 weeks (facility level)] / Total number of antenatal clients with a first contact	. Assessment of frequency for ANC attendance<br>. Identify the number and proportion of women who starts IPTp at the 13th week of pregnancy.<br>. Assess the IPTp3 uptake among PW<br>. Determine the prevalence of malaria among pregnant women in terms of number of episodes	CHW can check the reasons for not taking IPTp (Stock-out, Missing visit, …)
	*ANC 4 Attendance (%)		**Percentage of pregnant women with at least four ANC contacts** = [Number of pregnant women with four ANC contacts] / [Total number of antenatal clients with a first contact]		
	*ANC 8 Attendance (%)		**Percentage of pregnant women with a minimum of eight antenatal care contacts** = [Number of pregnant women with eight ANC contacts] / [Total number of pregnant women with a first contact]		
	IPTp3 Coverage (%)	Number of pregnant women who received IPTp3 / *Number of pregnant women received in ANC1*	**Percentage of women who received three doses or more of intermittent preventive therapy for malaria (IPTp) during their last pregnancy** = [Number of pregnant women given at least three doses of sulfadoxine–pyrimethamine for IPTp] / [Total number of antenatal clients with a first contact]		

	Counselling on danger signs (%)		**Pregnant women who received counselling on danger signs (%) during at least one ANC contact** = [Number of pregnant women who received counselling on danger signs / Total number of pregnant women with a first contact]		
	Behavioural counselling for the pregnant woman on how they individually prevent their exposure to malaria?				
	*ANC 2, ANC 3, ANC 4 and above loss to follow up rate (%)				
	IPTp 2 IPTp3, IPTp 4 and above loss to follow up rate (%)				
	Proportion of loss to follow-up (%)				
**Integrated vector malaria program**	LLIN distribution rate (%)	Number of LLINs routinely distributed to PW / *Number of PW received in ANC 1*		Determine the number and proportion of women who receive and effectively use the LLINs	. Assessing KAP of women regarding usage of LLINs (Check the instalment, usage, and maintenance of the LLINs<br>. Advice on good use and maintenance of LLINs)<br>. Identify via the DHIS 2 (Monthly Activity Report-MAR) the main communication channels used to inform PW on “How to use MN” (Health Care provider; CHW, Neighbour…)
	Routine LLIN performance (%)	Number of LLINs routinely distributed to pregnant women / *Number of LLINs to be routinely distributed during the period considered (performance framework*			
**Data management unit**	Confirmation rate (%)	[Number of cases of uncomplicated malaria with positive RDT + Number of cases of uncomplicated malaria with positive BS + Number of cases of severe malaria with positive RDT + Number of cases of severe malaria with positive BS] / *[Number of*		Proportion of women with fever (Fever is an emergency in pregnancy)<br><br>Clarify data related to malaria management	. Explain to PW the reasons why drugs are given<br>. Inform on the critical signs and symptoms to focus on.

		suspected cases of uncomplicated malaria + Number of suspected cases of severe malaria]		(suspected, confirmed, treated cases) and malaria prevention (LLINs, IPTp)<br><br>3. Treated cases: Number of tested women who received ACT	
	Test positivity rate (%)	[Number of positive RDTs + Number of positive blood smear (BS)] / *[Number of Rapid Diagnostic Tests (RDTs) performed by health personnel + Number of BS performed by health personnel]*			
	RDT positivity rate (%)	Number of positive RDTs/ *Number of Rapid Diagnostic Tests (RDTs) performed by health personnel*			
	BS positivity rate (%)	Number of positive thick drops / *Number of Blood Smear (BS) performed by health personnel*			
	Weight of RDTs in diagnosis (%)	[Number of Rapid Diagnostic Tests (RDTs) performed by health personnel] / *[Number of Rapid Diagnostic Tests (RDTs) performed by health personnel + Number of Blood Smear (BS) performed by health personnel]*			
	Diagnosis rate/Proportion of suspected cases having received a confirmatory examination (%)	[Number of Rapid Diagnostic Tests (RDTs) performed by health personnel + Number of Thick Drops (GE) performed by health personnel] / *[Number of suspected cases of uncomplicated malaria + Number of suspected cases of severe malaria]*			
	ACT prescription rate (%)	[Number of confirmed uncomplicated malaria cases treated by health personnel with the ASAQ combination + Number of confirmed uncomplicated malaria cases treated by health personnel with ACTs other than ASAQ] / *[Number of cases of uncomplicated malaria with positive RDT + Number of cases of*			

		uncomplicated malaria with positive BS]			
	Artesunate prescription rate (%)	Number of confirmed severe malaria cases treated with injectable artesunate / *[Number of cases of severe malaria with positive RDT + Number of cases of severe malaria with positive BS]*			
	ASAQ prescription rate (%):	Number of cases of confirmed uncomplicated malaria treated by health personnel with the ASAQ combination / *[Number of cases of uncomplicated malaria with positive RDT + Number of cases of uncomplicated malaria with positive BS]*			
	Injectable prescription rate (%)	[Number of confirmed severe malaria cases treated with injectable artesunate + Number of confirmed severe malaria cases treated with injectable artemether + Number of confirmed severe malaria cases treated with injectable quinine] / *[Number of cases of severe malaria with positive RDT + Number of cases of severe malaria with positive BS]*			
	Proportional morbidity (%)	[Number of cases of uncomplicated malaria with positive RDT + Number of cases of uncomplicated malaria with positive BS + Number of cases of severe malaria with positive RDT + Number of cases of severe malaria with positive BS] / *[Total number of consultations for illness (all causes combined)]*			
	Proportional morbidity (Suspected cases)	[Number of suspected cases of uncomplicated malaria + Number of suspected cases of severe malaria] /			

		Total number of consultations for illness (all causes combined)			
	Proportional mortality (%)	Number of people who died from confirmed malaria / *Total number of people who died from illness (all causes)*			
**Malaria Case management** *Discussing the needed indicators at the community level*	ANC 1 missing visit rate (%)	Number of pregnant women who did not start ANC, identified and referred by CHW to a health facility [30] / ………………………	N/A	. Developing the CHW application of BornFyne with Mapping option for geolocalisation and ease recovery of loss of view<br>. Planning of the distribution of IPTp from the 2nd dose<br>. Assessing the environmental malaria risk factors at community level : Hygiene and Sanitation risk factors<br>. Looking for the loss of view and identify reasons of the rupture of follow up<br>. Identifying the reasons for missing IPTp and/or ANC visit	***Counselling/Communication:**<br>1. Surveillance on services utilization;<br>2. IPTp follow up<br>3. Referral to the health centre (Suspected malaria cases, loss to follow up)<br>3. Surveillance on LLINs, and malaria cases<br>4. Malaria Risk Factors; Identifying environmental malaria risk factors (Mostly on hygiene and sanitation)<br>5. Performing RDT at community level<br>6. Filling the Monthly Activity report that is linked with the DHIS2 (MAR for community help workers still under review)
	ANC 2, ANC 3, ANC 4 and above loss to follow up rate (%)	*Number of pregnant women who missed ANC 2, ANC 3, ANC 4 and above) visit, identified and referred by CHW [30] / ……………………	N/A		
	IPTp 1 missing dose rate (%)		N/A		
	IPTp 2 IPTp3, IPTp 4 and above Coverage (%)	Number of pregnant women who received IPTp 2, IPTp3, IPTp 4 and above from the CHW [30] / ………………………..	N/A		
	Proportion of fever cases (fever?) detected by CHW (%)	Number of case of fever identified by CHW in the community [30] / ………………………	N/A		
	Proportion of disease case referred by CHW to health facility (%)		N/A		
	Percentage of fever cases tested by CHW (%)	Number of Rapid Diagnostic Tests (RDTs) performed and interpreted by Community Health Workers in cases of fever / *Number of cases of fever reported by Community Health Workers*	N/A		
	Number of IPTp distributed at community level	(Not yet defined, implemented in Cameroon)	N/A		
	Environmental hygiene and sanitation risk factors for malaria at community level: *Drinking water; Sanitation; Household composition, Education among women aged 15-*	Not yet defined	N/A		

	49 years, Cell phone ownership among women aged 15-49 years, Internet use among women [8].				
	Counselling on Danger signs at community level	Not yet defined	N/A		
	Behavioural counselling for the pregnant woman on how they individually prevent their exposure to malaria?	Not yet defined	N/A		

Abbreviations: *ACT- ANC- Antenatal Care; ANC 1- ASAQ-Artesunate-amodiaquine ; BS-Blood Smear ; CHW- Community Health Worker; DAK- Digital Adaptation Kit; DHIS2- Digital Health Information System 2 ; IND- Indicator; IPTp- Intermittent Preventive Treatment during Pregnancy; KAP- Knowledge Attitude Practice; LLIN-Long Lasting Insecticide Net ; MAR- Monthly Activity Report; MiP- Malaria in Pregnancy; PNMS- Prenatal Management System; PW- Pregnant Woman; RDT- Rapid Diagnostic Test; WHO- World Health Organization*

Note: Empty cells indicate the numerator, and denominator was not provided and will be defined by the team as part of the next steps

*Indicates it is present in DAK and the content has been integrated into BornFyne content with corresponding ICD codes. Any other element without an asterisk implies the team is yet to identify and define the questions and validate with the NCMP and test it before it is integrated into the BornFyne content
